# Intravenous Lipid Emulsions to Deliver Bioactive Omega-3 Fatty Acids for Improved Patient Outcomes

**DOI:** 10.3390/md17050274

**Published:** 2019-05-08

**Authors:** Philip C. Calder

**Affiliations:** 1 Human Development and Health, Faculty of Medicine, University of Southampton, Southampton SO16 6YD, UK; pcc@soton.ac.uk; 2 NIHR Southampton Biomedical Research Centre, University Hospital Southampton NHS Foundation Trust and University of Southampton, Southampton SO16 6YD, UK

**Keywords:** fish oil, omega-3, eicosapentaenoic acid, docosahexaenoic acid, inflammation, eicosanoid, cytokine, surgery, critical illness, parenteral nutrition

## Abstract

Lipids used in intravenous nutrition support (i.e., parenteral nutrition) provide energy, building blocks, and essential fatty acids. These lipids are included as emulsions since they need to be soluble in an aqueous environment. Fish oil is a source of bioactive omega-3 fatty acids (eicosapentaenoic acid and docosahexaenoic acid). Lipid emulsions, including fish oil, have been used for parenteral nutrition for adult patients post-surgery (mainly gastrointestinal). This has been associated with alterations in biomarkers of inflammation and immune defense, and in some studies, a reduction in length of intensive care unit and hospital stay. These benefits, along with a reduction in infections, are emphasized through recent meta-analyses. Perioperative administration of fish oil may be superior to postoperative administration, but this requires further exploration. Parenteral fish oil has been used in critically ill adult patients. Here, the influence on inflammatory processes, immune function, and clinical endpoints is less clear. However, some studies found reduced inflammation, improved gas exchange, and shorter length of hospital stay in critically ill patients if they received fish oil. Meta-analyses do not present a consistent picture but are limited by the small number and size of studies. More and better trials are needed in patient groups in which parenteral nutrition is used and where fish oil, as a source of bioactive omega-3 fatty acids, may offer benefits.

## 1. Introduction

**Figure 1:**
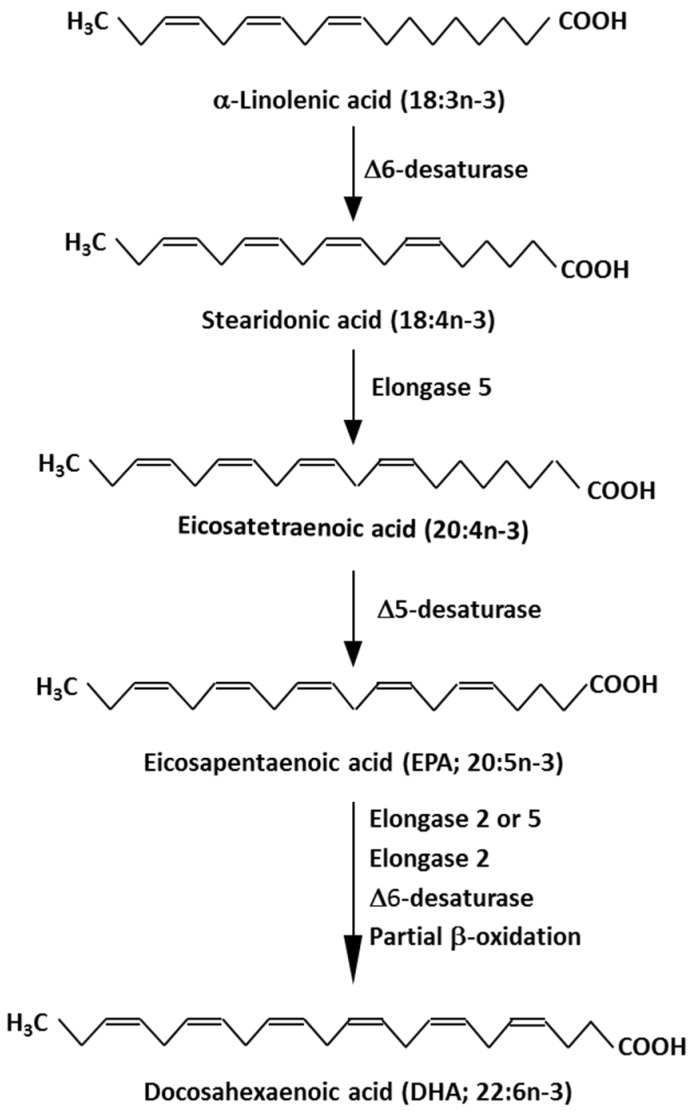
Pathway of biosynthesis of eicosapentaenoic acid (EPA) and docosahexaenoic acid (DHA) from precursor omega-3 (n-3) fatty acids.

Eicosapentaenoic acid (EPA, 20:5n-3) and docosahexaenoic acid (DHA, 22:6n-3) are biologically active long-chain omega-3 (n-3) polyunsaturated fatty acids [1,2]. EPA and DHA are produced from simpler n-3 fatty acids in a metabolic pathway involving sequential desaturation and elongation of the precursor fatty acids (Figure 1). For a variety of reasons, endogenous synthesis of EPA and DHA through this pathway is considered to be relatively poor in humans [3], placing a focus on intake of preformed EPA and DHA. Naturally rich sources of EPA and DHA include many marine organisms particularly fatty fish like salmon, trout, mackerel, herring, and sardines [4]. The body oils of fatty fish (and the liver oils of nonfatty (lean) fish like cod) can be isolated; these oils are rich in EPA and DHA although the content and relative amounts of EPA and DHA present are dependent upon the fish source [4]. These oils are generically termed “fish oils” and “fish liver oils” and are commonly used as dietary supplements. Other sources of EPA and DHA include krill oil and algal oils. For most people on a Western style diet, intake of EPA and DHA is low, but this can be increased markedly by eating fatty fish regularly or by using supplements which contain EPA and DHA [4]. When intake of EPA and DHA is increased, the amounts of those fatty acids in blood, blood cells, and tissues is increased [5,6,7].

**Figure 2:**
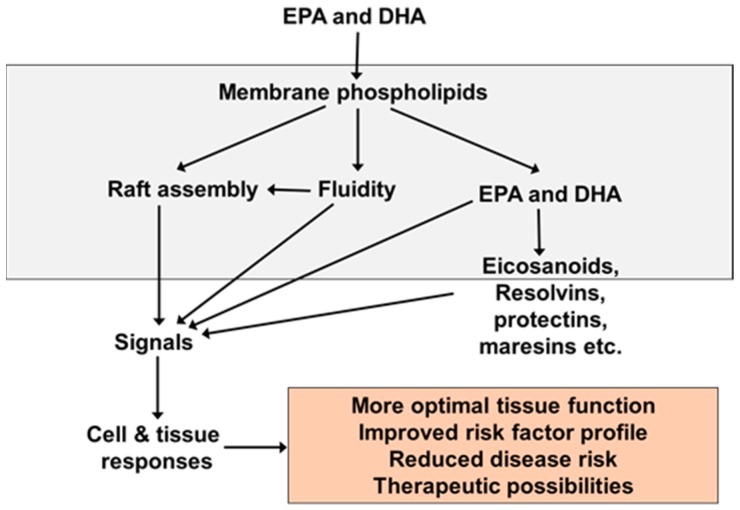
Generalized scheme of the mechanisms of action of eicosapentaenoic acid (EPA) and docosahexaenoic acid (DHA).

EPA and DHA are readily incorporated into the phospholipids of cell membranes and this is central to their biological activity (Figure 2), including their effects on inflammation and immune responses [8,9]. For example, they have been shown to modulate the physical characteristics of the membrane (termed membrane order or membrane fluidity) and the formation of signalling platforms called lipid rafts in many cell types, including in cells involved in inflammatory and immune responses [8,9]. These alterations in membrane structure and function have been shown to modify the signals generated at the membrane level that go on to influence cytosolic and nuclear events. For example, the ability of DHA to suppress phosphorylation of the inhibitory subunit of the proinflammatory transcription factor nuclear factor kappa B (NFκB) and to inhibit proinflammatory protein production in cultured macrophages in response to bacterial lipopolysaccharide (LPS) [10] was identified to be due to disruption of the formation of lipid rafts that occurs when the cells are exposed to LPS [11]. This observation creates a direct link between the incorporation of n-3 fatty acids into membranes, altered membrane responses to external stimuli, initiation of signalling cascades, gene expression, and protein production in inflammatory cells.

**Figure 3:**
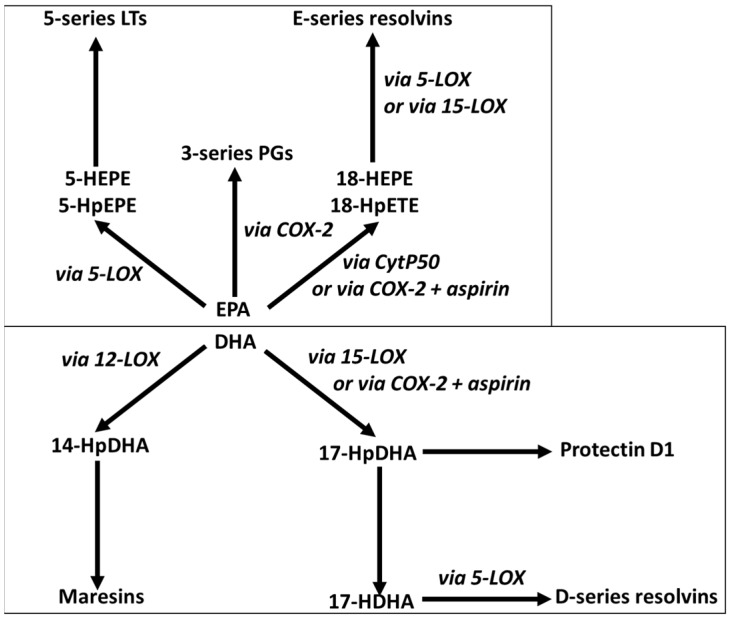
Overview of the pathways of conversion of eicosapentaenoic acid (EPA) and docosahexaenoic acid (DHA) to bioactive lipid mediators. EPA is metabolized via cyclooxygenase-2 (COX-2) to yield 3-series prostaglandins (PGs) and via 5-lipoxygenase (5-LOX) to yield 5-hydroperoxyeicosapenataenoic acid (HpEPE) which is converted to 5-hydroxyeicosapentaenoic acid (5-HEPE), the precursor of 5-series leukotrienes (LTs). EPA can also be metabolized to 18-HpETE by cytochrome P450 (CytP450) or by COX-2. In turn 18-HpEPE is converted to 18-HEPE which is metabolized by 5-LOX to resolvins E1 and E2 or by 15-lipoxygenase (15-LOX) to resolvin E3. DHA is metabolized via 12-lipoxygenase (12-LOX) to 14-hydroperoxydocosahexaenoic acid (14-HpDHA) which is converted to maresins. DHA can also be metabolized to 17-HpDHA by 15-LOX or by COX-2. 17-HpDHA is the precursor of protectin D1 and of 17-hydroxydocosahexaenoicacid (17-HDHA). 17-HDH is metabolized by 5-LOX to D-series resolvins. Different enantiomers of resolvins and protectins are produced in the absence or presence of aspirin.

EPA and DHA released from cell membrane phospholipids can be converted to bioactive lipid mediators through the action of cyclooxygenase, lipoxygenase, and cytochrome P450 enzymes (Figure 3). In this way, EPA and DHA are rather like the long-chain omega-6 (n-6) polyunsaturated fatty acid arachidonic acid (ARA, 20:4n-6), although the mediators produced from these three fatty acid substrates often have different biological activities or potencies [12,13].

EPA and DHA are incorporated into cell membranes at the expense of ARA, resulting in a shift in the pattern of the lipid mediators being produced. EPA, DHA, and their lipid mediator products also influence various transcription factors resulting in an altered expression of genes involved in many biological processes which include metabolism, immune function, and inflammation [9,12]. Consequently, through these actions from the membrane to the nucleus, EPA and DHA modify cell and tissue behavior and responses, and in general, these modifications are associated with more optimal function, an improved risk factor profile, a reduction in disease risk, and in some cases, therapeutic possibilities (Figure 2). EPA and DHA have long been recognized to have anti-inflammatory properties, including decreasing production of proinflammatory lipid mediators from ARA, decreasing production of key proinflammatory cytokines like tumor necrosis factor (TNF), interleukin (IL)-1β and IL-6, and reducing leukocyte-endothelium adhesion interactions [12,14]. More recently, EPA and DHA have been shown to be the precursors for potent inflammation resolving mediators termed resolvins, protectins, and maresins [15,16], which are produced through the pathways outlined in Figure 3. These molecules, collectively termed specialized pro-resolving mediators, have a range of potent actions including upregulating phagocytosis promoting clearance of damaged tissue and cellular debris and reducing production of classic inflammatory cytokines like TNF and IL-1β [15,16]. The combined actions of EPA and DHA suggest that they could be important in preventing, reducing the severity, and even treating chronic inflammatory conditions like rheumatoid arthritis [17,18,19]. Accumulation of EPA and DHA in cells and tissues from the diet or from oral supplements occurs over a time frame of days to weeks to months, depending upon the tissue involved [5,6,7]. In acute settings, more rapid delivery of EPA and DHA may be required. Lipid emulsions (LEs) that include fish oil as a source of EPA and DHA are commercially available for intravenous infusion as part of nutrition support for patients [20,21]. Intravenous administration of these LEs can quickly provide relatively high amounts of EPA and DHA if it is desired. This article will describe the rationale for the development of fish oil containing LEs and their application in surgical and critically ill patients.

## 2. Fish Oil Containing LEs for Intravenous Use

### 2.1. The Role of LEs in Intravenous Nutrition Support

It is not possible for some patients to consume food either transiently or in the longer term. If food intake beyond a few days is not possible, patients require what is termed “nutrition support” in order to maintain or restore optimal nutritional status and health. Nutrition support for patients should use the gastrointestinal tract whenever it is possible. However, there are instances where use of the gastrointestinal tract is not possible. These include patients with:

- a non-functional gastrointestinal tract due to:
  ○ surgical removal because of disease
  ○ intestinal blockage or leakage
  ○ impaired absorptive capacity
- severe gastrointestinal disease
- severe malnutrition
- trauma or critical illness

In such patients, the intravenous route should be used to provide nutrition support. This is referred to as parenteral nutrition. Parenteral nutrition should include a mix of macronutrients, as energy sources and substrates for biosynthesis, and micronutrients. It is important to include lipids as a component of parenteral nutrition. This is because the fatty acids within lipids are good sources of energy and reduce the need to provide large amounts of carbohydrates and they are building blocks for cell membranes required for tissue repair and host defenses. In addition, the provision of essential fatty acids is necessary to avoid a deficiency, which has been described in infants receiving long-term parenteral nutrition that was lipid free [22]. Finally, the fatty acids and the complex lipids that carry them may have bioactivities that affect the outcome for the patient [1,20,21]. In parenteral nutrition, lipids are provided as aqueous emulsions of oils that are mainly triglycerides, with a phospholipid monolayer which is usually phosphatidylcholine (lecithin) of soybean origin. A range of LEs are commercially available comprising various mixtures of soybean oil, oil rich in medium chain triglycerides (MCTs), olive oil, and fish oil. The composition of these LEs is summarized in Table 1.

### 2.2. Rationale for Fish Oil Containing LEs 

**Figure 4:**
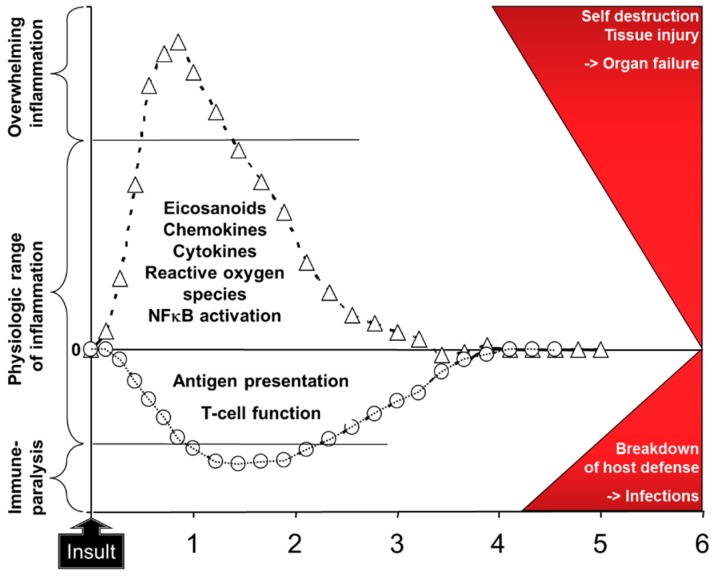
Schematic depiction of the response to insult with activation of inflammation and impairment of acquired immunity. It is considered that overwhelming inflammation and immune paralysis directly lead to adverse patient outcomes as depicted in the red area on the right. Examples of the “insult” include major surgery, wound or tissue injury, and the presence of infection. Modified from [27] with permission from Karger Publishers, Basel, Switzerland. Original figure Copyright © 2014, 2015 Karger Publishers, Basel, Switzerland.

As outlined above, EPA and DHA have a number of bioactivities [1,2,9,12,20,21]. Through these bioactivities EPA and DHA can affect metabolism, inflammation, immune responses, oxidative stress, blood coagulation, organ function (e.g., liver, lung, muscle, brain), and wound healing amongst others [1]. These effects are likely to be of relevance to patients receiving parenteral nutrition support [20,21]. In this regard there has been significant attention on the ability of EPA and DHA to modulate inflammation and the immune response. This is because of the increasing recognition that uncontrolled inflammation and a period of immune paralysis can occur in certain groups or subgroups of patients, sometimes concurrently, and that these are linked with poor patient outcomes such as increased risk of infections, longer stay in hospital, and in more seriously ill patients increased mortality. For example, patients undergoing gastrointestinal surgery showed elevated plasma concentrations of the inflammatory cytokine interleukin (IL)-6 in the hours to days following surgery, with higher concentrations observed in the more severely stressed patients [23]. At the same time, there was a decline in T lymphocyte function in those patients [23]. Patients in the early stages of sepsis showed higher concentrations of TNF, IL-1β, and IL-6 than healthy controls and they had an elevated activation of the proinflammatory transcription factor NFκB in blood leukocytes [24]. Both the inflammatory cytokines and the activation of NFkB were higher in the patients who did not survive than in survivors [24], suggesting an association between hyperinflammation and mortality. Bozza et al. [25] identified that the concentrations of some plasma cytokines measured at entry to the intensive care unit (ICU) predicted 48-hour and 28-day mortality in patients with sepsis. Andaluz-Ojeda et al. [26] studied 29 mainly elderly male patients with infections of whom 17 survived and 12 did not. They found that the blood concentrations of IL-6, IL-8, IL-10, and monocyte chemoattractant protein-1 were higher on the first day of admission to the ICU in non-survivors than in survivors, and that these cytokines were associated with mortality at days three and 28 after adjusting for disease severity at ICU entry. These authors reported that 28-day survival was over 90% in those patients with IL-6, IL-8, and IL-10 concentrations all < 75th percentile on day one, while survival was around 30% in those patients with IL-6, IL-8, and IL-10 concentrations all > 75th percentile on day one [26]. These observations suggest an important association between a strong inflammatory response and a poor outcome, perhaps mediated through organ damage and failure (Figure 4). Likewise, immune paralysis could lead to a poor outcome related to an increased susceptibility to infections (Figure 4).

**Figure 5:**
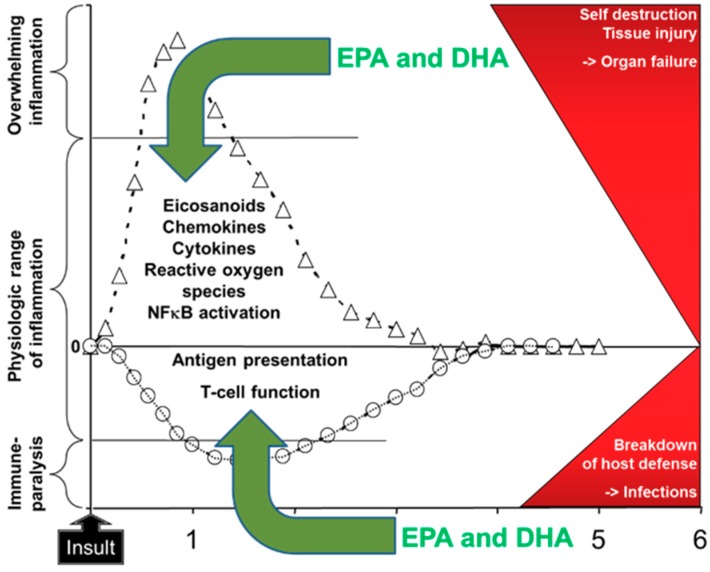
Rationale for inclusion of omega-3 fatty acids (eicosapentaenoic acid (EPA) and docosahexaenoic acid (DHA)) in intravenous nutrition support in patients at risk of or already displaying overwhelming inflammation and immune paralysis. Modified from [27] with permission from Karger Publishers, Basel, Switzerland. Original figure Copyright © 2014, 2015 Karger Publishers, Basel, Switzerland.

Using cell culture and animal models, the effects of EPA and DHA have been demonstrated on the following: eicosanoids like prostaglandin E_2_ and leukotriene B_4_; chemokines like monocyte chemoattractant protein 1; cytokines like TNF, IL-1β, IL-6, and IL-10; reactive oxygen species production; and NFκB activation as reviewed elsewhere [12,14]. Although less well explored, effects of EPA and DHA on the function of antigen presenting cells [28] and T cells [29] are also described in the literature. These effects provide a rationale for inclusion of fish oil in LEs used for nutrition support in patients undergoing major surgery or with critical illness (Figure 5). Preclinical models strongly support this approach. For example, fish oil decreases vasoconstriction, hypertension, and vascular permeability and leakage in an animal model of lung injury [30]; decreases the metabolic and inflammatory response to endotoxin, improving heart and lung function and survival [30,31,32]; and enhances survival in some models of infection [29]. The pro-resolving effects of EPA- and DHA-derived lipid mediators may also be relevant in this regard. For example, Spite et al. [33] reported that DHA-derived resolvin D2 reduced bacterial load in blood and peritoneum and improved survival in a caecal ligation and a puncture model of sepsis in mice. This was associated with much reduced plasma levels of several inflammatory cytokines (TNF, IL-1β, IL-6, IL-10, IL-17) and chemokines, reduced leukocyte infiltration into the peritoneum, and reduced peritoneal concentrations of prostaglandin E_2_ and leukotriene B_4_. In a murine model of sepsis induced by caecal ligation and puncture, DHA-derived resolvin D1 decreased the bacterial load in the blood and peritoneum, decreased lung injury, decreased plasma concentrations of TNF, IL-6, IL-10, and interferon-γ, and improved survival [34]. In parallel with these effects on inflammation, resolvin D1 decreased the proportion of T lymphocytes undergoing apoptosis [34].

### 2.3. Anti-Inflammatory and Immune Enhancing Effects of Fish Oil Containing LEs in Patients

**Figure 6:**
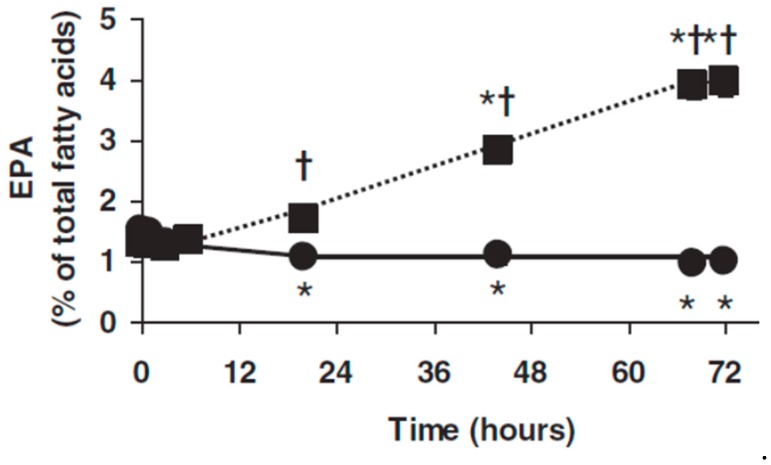
Plasma phosphatidylcholine eicosapentaenoic acid (EPA) in patients with hepatic colorectal metastases and receiving intravenous infusion of a blend of soybean oil, MCTs, and fish oil (closed squares) or soybean oil and MCTs (closed circles) daily for 72 h. * Indicates significantly different from study entry within the same group. † Indicates significant difference between groups at a given time point. Figure taken from Al Taan et al. [36].

LEs that include fish oil are an effective way of delivering EPA and DHA directly into the circulation [35,36,37]. Infusion of a blend of soybean oil, MCTs, and fish oil (50:40:10 vol/vol/vol) daily for five days in septic patients in the ICU who were intolerant of enteral feeding resulted in an elevation in the content of EPA, though not DHA, in plasma phosphatidylcholine at the end of the infusion period [35]. Patients with hepatic colorectal metastases requiring resection received continuous infusion of a blend of soybean oil, MCTs, and fish oil (50:40:10 vol/vol/vol) for 72 h prior to surgery [36]. EPA in plasma phosphatidylcholine was higher than at study entry, and higher than in the control group at 20, 44, 68 and 72 h (Figure 6) [36]. Barros et al. [37] infused a pure fish oil LE into ICU patients receiving enteral nutrition for 6 h on each of 3 consecutive days; blood samples were collected prior to the first infusion, and 24 and 72 h after the third infusion. There was a significant increased appearance of EPA and DHA in plasma phosphatidylcholine at the latter two time points as compared with both the study entry and the control group [37].

It has been estimated that a daily oral dose of 2–2.5 g of EPA plus DHA is required to elicit an anti-inflammatory effect in humans [38]. LEs are typically infused at a rate of up to 1 g lipid/kg body weight per day, and therefore a 70 kg patient could receive 70 g of LE if the emulsion was infused continuously over an entire day. Depending upon the exact LE used (Table 1) this would provide 70, 7 or 10.5 g fish oil daily for pure fish oil, fish oil blend 1, and fish oil blend 2, respectively. This amount of fish oil would supply about 27, 4 or 3.5 g EPA plus DHA daily from these three LEs. Clearly these amounts would differ according to a patient’s body weight, the LE infusion rate, and the infusion duration. Nevertheless, these figures indicate that anti-inflammatory doses of EPA and DHA can be delivered with LEs that are currently available. This is supported by the observations that using these fish oil containing LEs can decrease the blood concentrations or ex vivo production of proinflammatory eicosanoids [39,40] and cytokines [35,37,41] in surgical [39,40,41] and critically ill [35,37] patients. Randomized controlled trials (RCTs) of the effect of fish oil containing LEs on markers of inflammation and immune function in patients who had undergone surgery for gastrointestinal cancers were subject to a very recent meta-analysis [42]. Depending upon the biomarker, the analysis included between four and 13 RCTs and between 209 and 756 patients. It was determined that fish oil LEs resulted in significant decreases in the inflammatory markers TNF, IL-6, and C-reactive protein (all *P* < 0.00001) and significant increases in markers of acquired immunity including the numbers of lymphocytes (*P* < 0.0001), CD3 and CD4 cells and the CD4 to CD8 ratio (all *P* < 0.00001), and the concentrations of immunoglobulins A, M, and G (all *P* < 0.00001) [42]. These observations support the proposal shown in Figure 5 that fish oil containing LEs can be used to control inflammation and support immune function in patients receiving parenteral nutrition.

## 3. Clinical Studies in Patients Undergoing Surgery

According to Figure 5, better control of inflammation and better support of immune defenses would be linked with improved patient outcomes. Section 2.3 describes that the use of fish oil containing LEs significantly decreases markers of inflammation and significantly increases markers of acquired immune defenses, especially in surgical patients. Therefore, it would be expected that fish oil LEs would improve patient outcomes in surgical patients. Whether this is the case has been mainly explored in patients undergoing gastrointestinal surgery usually for the removal of malignant tissue. LEs have mainly been used in the days immediately following surgery (usually days 1 to 5) although there are a small number of trials of longer duration or using perioperative administration. The clinical outcomes most often reported are infections and length of hospital stay, while length of ICU stay where patients went to the ICU post-surgery is also reported. Individual trials are discussed in detail elsewhere [20,21]. There have been a number of meta-analyses of the studies conducted with fish oil LEs in surgical patients [42,43,44,45,46,47]. These meta-analyses are summarized in Table 2. The findings of these meta-analyses are consistent and it is evident that compared with other LEs, usually based on pure soybean oil or a 50:50 (vol/vol) blend of soybean oil and MCT oil, fish oil containing LEs can decrease infections, the length of ICU stay, and the length of hospital stay in surgical patients. As indicated above, most of the studies have used LEs postoperatively but it would also seem advantageous to use fish oil containing LEs preoperatively for several days in cases of elective surgery in order to get the bioactive omega-3 fatty acids into the body in advance of the surgical insult.

## 4. Clinical Studies in Patients Requiring Critical Care

There are fewer studies that compare different intravenous LEs in critically ill patients to in surgical patients, yet critically ill patients are more likely to suffer the adverse effects of hyperinflammation and immune paralysis, are more likely to have poor outcomes like organ failure and death, are more likely to have a prolonged hospital stay, and are more likely to require nutrition support including parenteral support. The trials that have been performed most often report infections, respiratory function and need for ventilator support, length of ICU and hospital stay, and death. Individual trials of fish oil containing LEs in critically ill patients are reviewed in detail elsewhere [20,21,27,48,49]. There have been several meta-analyses of the studies conducted with fish oil LEs in critically ill patients [45,50,51,52]. These meta-analyses are summarized in Table 3. The outcomes of these meta-analyses reflect the mixed picture that emerges from the different trials, that is to say, different meta-analyses produce different findings for some outcomes. This may reflect the relatively small number of studies performed, the use of different fish oil containing LEs, and the heterogeneity of this patient population. Thus, at this stage it is difficult to make a conclusive statement about the role of fish oil containing LEs in critically ill patients, although the rationale, as depicted in Figure 5, remains highly relevant.

**Figure 7:**
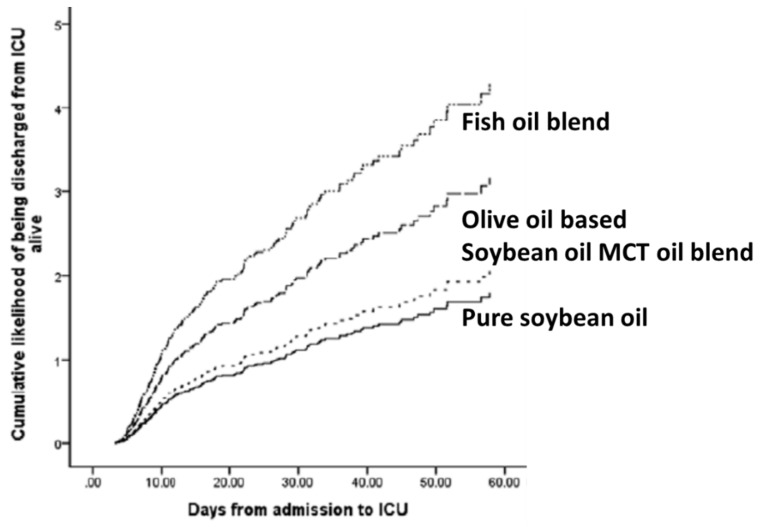
Cumulative likelihood of critically ill patients being discharged from the ICU alive according to the LE received. Modified with permission from C.E. Edmunds, R.A. Brody, J.S. Parrott, S.M. Stankorb, D.K. Heyland (2014) The effects of different IV fat emulsions on clinical outcomes in critically ill patients. Critical Care Medicine 42, 1168–1177 [53].

Moving away from RCTs and closer to the real patient setting, Edmunds et al. [53] published an interesting secondary analysis of data from a prospective multicenter international study. The study included adults admitted to the ICU for more than 72 h and who were ventilated within 48 h. To be included in the secondary analysis, patients had to have received parenteral nutrition exclusively for more than 5 days and to have received a single type of LE during that time. Of the available 12,585 patients only 451 (3.5%) met these criteria, most (84.2%) having received enteral nutrition. Among the 451 patients included, 223 (49.4%) received pure soybean oil LE while only 19 (4.2%) received a LE that included fish oil. The findings of the study are summarized in Table 4. As compared with using pure soybean oil LE or a 50:50 blend of soybean oil and MCTs, use of fish oil was associated with fewer patient deaths by day 60, shorter lengths of ICU stay, and shorter lengths of hospital stay. Figure 7 shows that the use of fish oil containing LEs increased the likelihood of a patient being discharged alive from the ICU. These findings from a prospective study rather than from an RCT are indicative of a significant clinical benefit from fish oil LEs in critically ill patients. However, it needs to be recognized that the number of patients receiving intravenous fish oil was very small (4.2% of those receiving exclusive parenteral nutrition with one LE and only 0.15% of the entire patient cohort). Thus, the findings must be considered cautiously. Furthermore, it is possible that those centers that use fish oil LEs may use other innovative approaches that benefit their patients. Nevertheless, these findings are encouraging and support the design of improved trials for the future.

## 5. Summary and Conclusions

Lipids used in intravenous nutrition support (i.e., parenteral nutrition) provide energy, building blocks, and essential fatty acids. These lipids are included as emulsions since they need to be soluble in an aqueous environment. Fish oil is a source of bioactive omega-3 fatty acids (EPA and DHA) in contrast to the more traditional soybean oil which is rich in the omega-6 fatty acid linoleic acid. Preclinical research suggests that including fish oil in parenteral nutrition support may control adverse inflammatory responses and may support acquired immunity, thereby offering advantages to patients that would be seen through improved clinical outcomes. LEs, including fish oil, have been used for parenteral nutrition for adult patients post-surgery (mainly gastrointestinal). This has been associated with alterations in patterns of inflammatory mediators and in immune function, and in some studies, a reduction in length of ICU and hospital stay. These benefits, as well as a reduction in infections, are brought out by recent meta-analyses. Perioperative administration of fish oil may be superior to postoperative, but this requires greater exploration. Parenteral fish oil has been used in critically ill adults. Here, the influence on inflammatory processes, immune function, and clinical endpoints is not clear, because there are too few studies and those that are available report inconsistent findings. However, some studies found reduced inflammation, improved gas exchange, and shorter length of hospital stay in critically ill patients if they received fish oil. Meta-analyses do not provide a clear picture of the impact of fish oil containing LEs in critically ill patients, but these are limited by the small number and size of studies performed so far. A prospective study suggests a benefit from fish oil LEs in critically ill patients but in that study very few patients received fish oil. More and better trials are needed in patient groups in which parenteral nutrition is used and where fish oil may offer benefits.

## Figures and Tables

**Table 1:** Oil sources and major fatty acids (% of total) of commercially available lipid emulsions for use in parenteral nutrition.

	Pure Soybean Oil	Soybean Oil MCT Oil Blend	Restructured Soybean Oil MCT Oil Blend	Pure Fish Oil	Olive Oil Based	Fish Oil Blend 1	Fish Oil Blend 2
Oil source (%):							
Soybean	100	50	64	-	20	40	30
MCT	-	50	36	-	-	50	30
Olive	-	-	-	-	80	-	25
Fish	-	-	-	100	-	10	15
Fatty acids (%)							
Saturated	15	58	46	21	14	49	37
Monounsaturated *	24	11	14	23	64	14	33
Polyunsaturated	61	31	40	56	22	37	30
Omega-3	8	4	5	48	3	10	7
ALA	8	4	5	1	3	4	2
EPA	-	-	-	20	-	3.5	3
DHA	-	-	-	19	-	2.5	2
Omega-6 **	53	27	35	5	19	27	23

ALA, α-linolenic acid; EPA, eicosapentaenoic acid; DHA, docosahexaenoic acid. * mainly oleic acid (18:1n-9). ** Mainly linoleic acid (18:2n-6). Note that the fatty acid composition of fish oil is more variable than that of vegetable oils so that the precise contribution of different fatty acids may differ in different batches.

**Table 2:** Summary of meta-analyses of randomized controlled trials of fish oil containing lipid emulsions (LEs) in surgical patients.

	Effect of Fish Oil LE On		
Meta-Analysis and Year	Infections	Length of ICU Stay	Length of Hospital Stay
Chen et al. (2010) [43]	Odds ratio 0.56 (0.32, 0.98) *P* = 0.04 *n* = 7 studies	−1.80 days (−3.04, −0.56) *P* = 0.004 *n* = 5 studies	−2.98 days (−4.65, −1.31) *P* = 0.0005 *n* = 7 studies
Wei et al. (2010) [44]	Risk ratio 0.49 (0.26, 0.93) *P* = 0.03 *n* = 4 studies	−2.07 days (−3.47, −0.47) *P* = 0.004 *n* = 3 studies	
Pradelli et al. (2012) [45] (non-ICU patients)	Risk ratio 0.53 (0.34, 0.82) *P* = 0.004 *n* = 6 studies		−1.86 days (−3.13, −0.59) *P* = 0.0004 *n* = 6 studies
Li et al. (2014) [46]	Odds ratio 0.53 (0.35, 0.81) *P* = 0.003 *n* = 9 studies		−2.14 days (−3.02, −1.27) *P* < 0.00001 *n* = 11 studies
Bae et al. (2017) [47]	Odds ratio 0.44 (0.30, 0.65) *P* < 0.0001 *n* = 15 studies		−2.70 days (−3.60, −1.79) *P* < 0.00001 *n* = 10 studies
Zhao and Wang (2018) [42]	Odds ratio 0.36 (0.20, 0.66) *P* = 0.0008 *n* = 8 studies		

**Table 3:** Summary of meta-analyses of randomized controlled trials of fish oil containing lipid emulsions (LEs) in critically ill patients.

	Effect of Fish Oil LE On				
Meta-Analysis and Year	Infections	Length of ICU Stay	Length of Hospital Stay	Ventilation Requirement	Mortality
Pradelli et al. (2012) [45] (ICU patients)	Odds ratio 0.71 (0.45, 1.12) *P* = 0.14 *n* = 5 studies	−1.92 days (−3.27, −0.58) *P* = 0.005 *n* = 8 studies	−5.17 days (−8.35, −1.99) *P* = 0.001 *n* = 8 studies		
Palmer et al. (2013) [50]	Risk ratio 0.78 (0.43, 1.41) *P* = 0.41 *n* = 5 studies	−0.57 days (−5.05, 3.90) *P* = 0.80 *n* = 6 studies	−9.49 days (−16.51, −2.47) *P* = 0.008 *n* = 3 studies		Risk ratio 0.83 (0.57, 1.20) *P* = 0.32 *n* = 8 studies
Manzanares et al. (2014) [51]	Risk ratio 0.76 (0.42, 1.36) *P* = 0.35 *n* = 3 studies	−1.13 days (−8.96, 6.69) *P* = 0.78 *n* = 3 studies		−1.81 days (−3.98, 0.36) *P* = 0.10 *n* = 3 studies	Risk ratio 0.71 (0.49, 1.04) *P* = 0.08 *n* = 5 studies
Manzanares et al. (2015) [52]	Risk ratio 0.64 (0.44, 0.92) *P* = 0.02 *n* = 5 studies	−1.42 days (−4.53, 1.69) *P* = 0.37 *n* = 7 studies	−3.71 days (−9.31, 1.88) *P* = 0.19 *n* = 7 studies	−1.14 days (−2.67, 0.38) *P* = 0.14 *n* = 6 studies	Risk ratio 0.90 (0.67, 1.20) *P* = 0.46 *n* = 9 studies

**Table 4:** Summary of findings of Edmunds et al. [53].

Outcome	Soybean Oil	Soybean Oil MCT Oil Blend	Fish Oil Blend
Patient died within 60 days (%)	28.3	30.8	10.5
Duration of mechanical ventilation (median days)	4.9	5.3	5.0
Length of ICU stay (median days)	10.9	9.6	7.05
Length of hospital stay (median days)	28.1	31.9	14.1
